# Changes in Drug Utilization After Publication of Clinical Trials and Drug-Related Scandals in Japan: An Interrupted Time Series Analysis, 2005–2017

**DOI:** 10.2188/jea.JE20200181

**Published:** 2021-07-05

**Authors:** Shingo Fukuma, Tatsuyoshi Ikenoue, Yukari Yamada, Yoshiyuki Saito, Joseph Green, Takeo Nakayama, Shunichi Fukuhara

**Affiliations:** 1 Human Health Sciences, Kyoto University Graduate School of Medicine, Kyoto, Japan; 2 Research Division, Institute for Health Outcomes and Process Evaluation Research (iHope International), Tokyo, Japan; 3 Department of Health Informatics, Kyoto University Graduate School of Medicine and School of Public Health, Kyoto, Japan; 4 Section of Clinical Epidemiology, Department of Community Medicine, Kyoto University, Kyoto, Japan; 5 Center for Innovative Research for Communities and Clinical Excellence, Fukushima Medical University, Fukushima, Japan; 6 Shirakawa STAR for General Medicine, Fukushima Medical University, Fukushima, Japan

**Keywords:** clinical trial, drug utilization, research scandal, interrupted time series analysis

## Abstract

**Background:**

Breaches of ethics undermine the practice of medicine. In Japan, two major scandals involving clinical research and drug marketing occurred after the publication of clinical trials. To study the effects of those scandals, we evaluated changes in the use of first-generation angiotensin II receptor blockers (ARBs) after publication of relevant clinical trials and also after the subsequent scandals.

**Methods:**

We conducted a quasi-experimental design of an interrupted time series analysis (ITSA) on nationwide monthly drug-market data covering 12 years (2005 to 2017) in Japan. The main outcome was the use of first-generation ARBs (valsartan, candesartan, and losartan). The two exposures were the publication of ARB-related clinical-trial results (October 2006) and subsequent ARB-related scandals involving research and marketing (February 2013). A generalized estimating equation model was fitted for ITSA with a log link, Poisson distribution, robust variance estimators, and seasonality adjustment.

**Results:**

The publication of clinical trials was associated with 12% increase in the use of first-generation ARBs in Japan, and the subsequent ARB-related scandals was associated with 19% decrease. The decrease in the use of first-generation ARBs after the scandals was greater than the increase in their use after the publication of clinical-trial results. The net effect of the two exposures was a 9% decrease in the use of first-generation ARBs.

**Conclusions:**

The scandals were associated with decrease in the use of first-generation ARBs, and that decrease was greater than the increase associated with the publication of “successful” clinical trials, making the net effect not zero but negative.

## INTRODUCTION

When medical research adheres to ethical norms, it is reasonable for the results of that research to affect clinical practice.^1^^–^^5^ Positive clinical-trial results published in prestigious journals are likely to be trusted by physicians, and physicians use trusted evidence when they make clinical decisions. In addition, those results are used by pharmaceutical companies to increase the sales of their products.^3^ Pharmaceutical-related scandals that bring the validity of clinical trials into question might have the opposite effect on clinical-practice decisions and thus could decrease sales of the drugs concerned.

In Japan, scandals caused by breaches of ethics in medical research and pharmaceutical marketing occurred following the publication of the results of clinical trials of first-generation angiotensin II receptor blockers (ARBs).^6^^–^^8^ ARBs have been widely used in Japan as antihypertensive agents since the introduction of first-generation ARBs (valsartan, candesartan, and losartan) in 1999–2000. In October 2006, the results of large post-marketing Japanese clinical trials of first-generation ARBs were presented at the 21^st^ scientific meeting of the International Society of Hypertension, in Fukuoka, Japan. Pharmaceutical companies made extensive use of those results in promoting their first-generation ARBs as superior to other antihypertensive drugs. The results of the JIKEI-HEART and the CASE-J studies were published in *The Lancet* in April 2007^9^ and in *Hypertension* in February 2008,^10^ respectively. The following year, the results of another Japanese clinical trial, called KYOTO-HEART, were published in the *European Heart Journal*.^11^ However, by 2012, concerns about the validity of the results of those trials had become public.^12^ The articles reporting the results of the JIKEI-HEART and KYOTO-HEART studies, as well as three related articles (sub-analyses of data from the KYOTO-HEART study), were retracted because data had been fabricated and falsified.^13^^–^^18^ An official report on this research fraud was published by Japan’s Ministry of Health, Labour and Welfare in October 2013.^19^ A major scandal ensued and the incident was widely reported in Japanese mass media. The intensity of the public response was due in no small part to the fact that approximately $3 billion United States dollars, much of it public money, was being spent each year on first-generation ARBs in Japan.^19^^–^^21^ At the same time, an important marketing-related pharmaceutical scandal erupted when it was revealed that promotional activities using results of the CASE-J study were misleading.^7^^,^^22^^,^^23^ In June 2015, the Japanese government issued a business improvement order based on the Pharmaceutical Affairs Law, saying that drug-promotion materials using the CASE-J study’s results were deceptive. Conflicts of interest in various studies of first-generation ARBs have also been documented.^8^ In April 2017, the Japanese government enacted a law to prevent such problems from recurring and thus to restore the public’s trust in clinical research (the Clinical Trials Act).^24^

These events can be seen as a natural experiment of population-level interventions, making it possible to quantitatively assess the impact of positive clinical-trial results and the impact of subsequent research-related and marketing-related scandals by a quasi-experimental design, as regards drug-prescribing practices. Thus, we studied nationwide drug-utilization data that were collected monthly in Japan between April 2005 and March 2017. Using an interrupted time series analysis (ITSA), we assessed how the use of first-generation ARBs changed after the trials’ results were published, and how it changed again after the scandals occurred.

## METHODS

### Design, setting, and data source

This was a population-based analysis of an ITSA^25^^,^^26^ using nationwide drug-market records in the IQVIA IMSBase Japan Pharmaceutical Market (JPM) database. The records used covered the period between April 2005 and March 2017. During that period, Japan had a population of approximately 127.5 million, among whom approximately 10 million received medical care for hypertension.^27^ Under Japan’s universal healthcare system, physicians can prescribe any drug as long as they comply with the usage and dosage regulated by health-insurance laws.^28^ The JPM database includes drug-sales data that were collected through wholesalers all over Japan. In Japan, wholesalers purchase and distribute most prescription drugs to medical facilities and pharmacies. Therefore, the JPM database covers 99% of insurance-covered drugs in Japan and contains information on the name, form, and country-level utilization dose of drug products. We estimated the usage of drugs from sales data in the JPM database. Records regarding antihypertensive drugs (eTable 1) were extracted.

### Use of first-generation ARBs

The main outcome was the use of first-generation ARBs (valsartan, candesartan, and losartan). Specifically, we began with the defined daily dose [DDD] per 1,000 persons. Then, for each month from April 2005 to March 2017, we computed the number of patients who received the drugs, that is, the country-level aggregated drug dose divided by the DDD (eTable 1).^29^ The DDD is “the average maintenance dose of the drug” and is approved by the World Health Organization (WHO). To calculate the numbers of patients receiving the drugs per 1,000 persons, we used the total number of residents of Japan from census data.^27^ Patients receiving angiotensin-converting-enzyme (ACE) inhibitors comprised the control group.

### Period of exposures to clinical trial publications and to subsequent scandals

Two exposures were considered: first, in October 2006 the results two clinical trials of first-generation ARBs (the JIKEI-HEART study and the CASE-J study) were presented at the conference of the International Society of Hypertension. Over the following 3 years, the results of the JIKEI-HEART study,^9^ the CASE-J study,^10^ and the KYOTO-HEART study^11^ were published in, respectively, *The Lancet*, *Hypertension*, and the *European Heart Journal*. Second, research fraud in the aforementioned studies was revealed, and a series of retractions of published results began in February of 2013. Deceptive marketing of Candesartan was also exposed in 2013.^23^

The publications and scandals that comprised the exposures are summarized in Table 1. Considering that the effects of those exposures on clinical practice would not have been immediate, we set a 6-month lag for each exposure. Other clinical trials of first-generation ARBs in Japan are summarized in eTable 2.

**Table 1.** Clinical trials of first-generation ARBs in Japan that were followed by scandals

Trial name	Trial’s purpose and conclusion	Publications, promotional use, and scandals
**Jikei Heart**^a^	In this trial, the researchers aimed to examine the effect of ***valsartan*** on cardiovascular disease in patients with hypertension and coronary heart disease or heart failure. The researchers wrote that “the addition of valsartan to conventional treatment prevented more cardiovascular events than supplementary conventional treatment.”	**Publication:** The results were presented at ISH2006,^d^ and they were published in *The Lancet* in April 2007.
		**Promotion:** After ISH2006, the results were used for commercial promotional activities.
		**Scandal:** Because of fabricated data, the published report was retracted in September 2013.

**Case-J**^b^	In this trial, the researchers aimed to examine the effect of ***candesartan*** on cardiovascular disease in high-risk patients with hypertension. The researchers wrote that “candesartan-based and amlodipine-based regimens produced no statistical differences in terms of the primary cardiovascular end point, whereas candesartan prevented new-onset diabetes more effectively than amlodipine.”	**Publication:** The results were presented at ISH2006, and they were published in *Hypertension* in February 2008.
		**Promotion:** After ISH2006, the results were used for commercial promotional activities.
		**Scandal:** Because of misuse of the trial’s results in commercial promotional activities, the government of Japan issued a business improvement order to the pharmaceutical company in May 2016.

**Kyoto Heart**^c^	In this trial, the researchers aimed to examine the effect of ***valsartan*** on cardiovascular disease in patients with uncontrolled hypertension. The researchers wrote that “valsartan add-on treatment to improve blood pressure control prevented more cardiovascular events than conventional non-ARB treatment in high-risk hypertensive patients in Japan.”	**Publication:** The results were presented at ESC2009,^e^ and they were published in the *European Heart Journal* in October 2010.
		**Promotion:** After ESC2009, the results were used for commercial promotional activities.
		**Scandal:** Because of fabricated data, the published report was retracted in February 2013.

^a^Jikei Heart: Valsartan in a Japanese population with hypertension and other cardiovascular disease.

^b^CASE-J: Candesartan Antihypertensive Survival Evaluation in Japan.

^c^Kyoto Heart: Effects of valsartan on morbidity and mortality in uncontrolled hypertensive patients with high cardiovascular risks.

^d^ISH2006: The 21st Scientific Meeting of the International Society of Hypertension, which was held in October of 2006, in Fukuoka, Japan.

^e^ESC2009: The congress of the European Society of Cardiology that was held in September of 2009 in Barcelona, Spain.

To assess the timing and the magnitude of the publicity given to the exposures and to the scandals in Japan, we searched Nikkei Telecom, a commercial database that covers news releases and many Japanese newspapers. The search terms for the trials were “JIKEI-HEART, CASE-J, or KYOTO-HEART” in Japanese. The search terms for the scandals were “(valsartan or candesartan or ARB) and (research fraud, or conflict of interest, or data manipulation, or data falsification, or hype)” in Japanese. Using the results of those searches, we counted the number of reports of the clinical trials, and the number of reports of the scandals, per quarter from April 2005 through March 2017.

### Statistical analysis

To assess how the two exposures (the publications and the subsequent scandals) affected the use of first-generation ARBs, we conducted a quasi-experimental design of an ITSA^25^^,^^26^^,^^30^ of the monthly data on drug utilization. In ITSA, we modeled two types of changes in the use of first-generation ARBs after the exposures: changes in the “level” of the use of first-generation ARBs (ie, DDDs/1,000 persons) and changes in the “slope” (ie, DDDs/1,000 persons/month). A generalized estimating equation (GEE) model^31^ was fitted with a log link, Poisson distribution, robust variance estimators, and identical covariance matrix, using the log-transformed total number of residents in Japan as the offset variable. To adjust for seasonality, we included dummy variables of calendar months in the model. A scale parameter to correct for overdispersion was also included. For comparison, we also modeled changes in the use of a different type of antihypertensive drug: ACE inhibitors. The ITSA model is described in detail in eMaterials 1. Next, we simulated the number of patients receiving first-generation ARBs in a counterfactual situation, supposing that no scandals had occurred. To model that situation, the effect of scandals in the post-scandal period was removed from the equation.

Five ITSA models of the association between exposures and outcomes are described below. After developing model #1, we tested its fit to the data relative to the fit of four other models (#2 though #5 described below). That testing was done by computing the quasi-likelihood under the independence model criterion (QIC)^32^ for each model. Lower values of the QIC indicate better fit. The five models were: 1) changes in level and changes in slope, with seasonality adjustment via calendar-month indicators, correlation structure: independent; 2) changes in level and changes in slope, with seasonality adjustment via calendar-month indicators, correlation structure: exchangeable; 3) changes in level and changes in slope, with seasonality adjustment via calendar-month indicators, correlation structure: autoregressive; 4) changes in level only (ie, no changes in slope), with seasonality adjustment via calendar-month indicators, correlation structure: independent; 5) changes in level and changes in slope, with seasonality adjustment via Fourier transformation, correlation structure: independent (eTable 3). In a supplementary analysis, we changed the comparison group from ACE inhibitors to calcium channel blockers (eMaterials 2). In another supplementary analysis, we assessed change in use of second-generation ARBs (eFigure 1). The method and reporting of the ITSA were assessed using a published checklist of recommendations,^26^ and details of compliance with those recommendations are in eTable 4. All analyses were conducted with Stata version 15.1 (StataCorp, College Station, TX, USA). All tests were two-sided, with *P* values less than 0.05 considered statistically significant.

### Institutional review board approval

Because this was an observational study of an aggregated dataset containing no information by which any individual could be identified, the institutional review board process was waived.

## RESULTS

**Figure 1.**
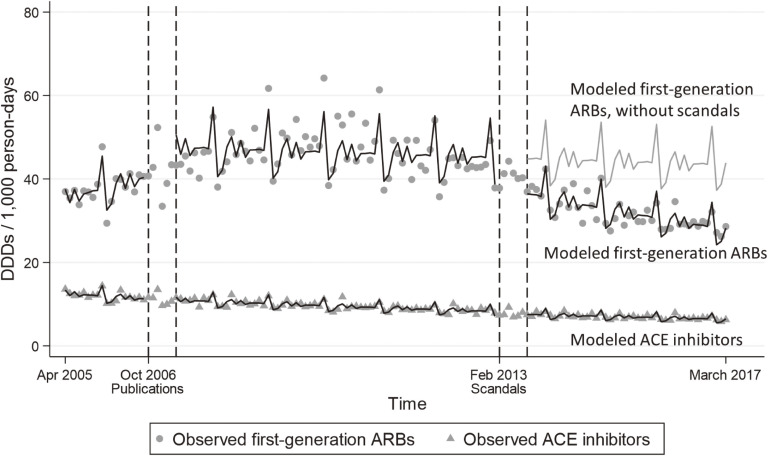
Changes in the use of first-generation ARBs and ACE inhibitors. This figure shows changes in the use of first-generation ARBs and ACE inhibitors (DDDs/1,000 persons) before and after the publication of clinical-trial results, and also before and after the occurrence of the scandals. The black lines indicate the modeled values for the use of first-generation ARBs and ACE inhibitors, according to the generalized estimating equation that is shown in eMaterials 1, which includes adjustment for seasonality. The grey line indicates the modeled values for the use of first-generation ARBs, supposing that there had been no scandals. The transient increase at the end of every calendar year is most likely caused by hospitals buying drugs just before the long new-year’s holiday, and it appears to be offset by a transient decrease of similar magnitude at the start of the following calendar year.

Changes in the levels and slopes of the use of first-generation ARBs after publication of clinical-trial results and after occurrence of the scandals can be seen in Figure 1. Publication of the clinical-trial results was associated with an increase in the use of first-generation ARBs (relative change in DDDs/1,000 persons, 1.12; 95% confidence interval [CI], 1.10**–**1.14). In contrast, the scandals were associated with a decrease (relative change in DDDs/1,000 persons, 0.81; 95% CI, 0.81**–**0.81). Before the results of the clinical trials were published, the use of first-generation ARBs had been increasing (annual change in DDDs/1,000 persons/year, 1.10; 95% CI, 1.08**–**1.11). There was little change between the time of the trials’ publication and the scandals (annual change in DDDs/1,000 persons/year, 0.99; 95% CI, 0.99**–**0.99). Once the scandals erupted, the use of first-generation ARBs decreased (annual change in DDDs/1,000 persons/year, 0.93; 95% CI, 0.93**–**0.93). The net effect of the two exposures was a 9% decrease in the use of first-generation ARBs (relative change in DDDs/1,000 persons, 0.91; 95% CI, 0.80**–**0.93).

In the simulated counterfactual situation (ie, using an equation that did not include a term for the scandals), the use of first-generation ARBs was greater than in the actual data by a median of 4.31 DDDs/1,000 persons (interquartile range, 1.55–12.23), which is equivalent to more than half a million people in Japan at that time. Specifically, we estimated that in Japan the scandals resulted in first-generation ARBs being used by 547,000 fewer people.

**Figure 2.**
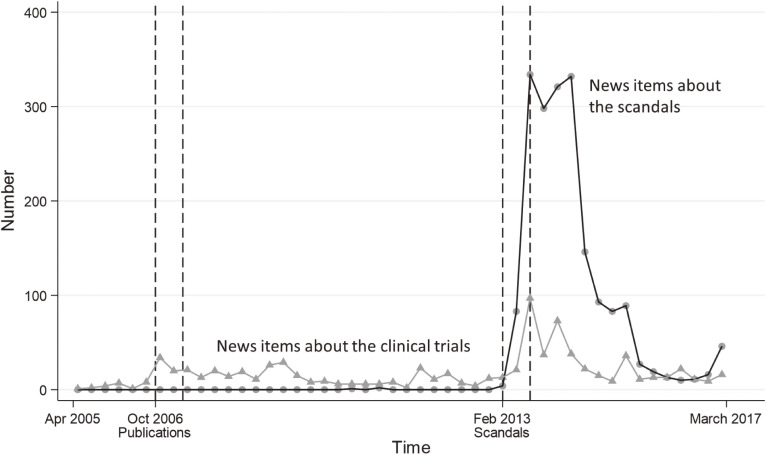
Mass-media coverage of clinical trials and the subsequent scandals. This figure shows the number of news items reporting on the clinical trials and on the scandals. Quarterly data covering the period from April 2005 through March 2017 were obtained from a commercial database that includes news releases and many Japanese newspapers. The search terms for the trials were “JIKEI-HEART, CASE-J, or KYOTO-HEART” in Japanese. The search terms for the scandals were “(valsartan or candesartan or ARB) and (research fraud, or conflict of interest, or data manipulation, or data falsification, or hype)” in Japanese.

When the results of the trials were first made public they were reported in Japanese news media (Figure 2). Once the scandals erupted, the number of news items about the clinical trials and about the scandals increased quickly. The number of news items about the trials just after their results were published was far exceeded by the number of news items about the scandals (Figure 2).

On the basis of QIC, the best model was the one that included changes in level and changes in slope, with seasonality adjustment via calendar-month indicators (eTable 3). When the comparison group was changed to calcium channel blockers, the results were similar to those of the main analysis (eTable 4).

## DISCUSSION

Using nationwide drug-market data, this study shows how the use of first-generation ARBs changed after the trials’ results were published, and how it changed again after the scandals occurred. The publication of apparently-successful clinical trials was followed by an increase in the use of first-generation ARBs, and the subsequent research-related and marketing-related scandals were followed by a decrease. Together, those two exposures were associated with a 9% decrease in ARB use. These results show the magnitude of the impact of breaches of trust on clinical practice.

In Japan, clinical guidelines have recommended ARBs as first-line drugs for treating hypertension since 2001,^33^ which might have caused the increase in prescriptions of first-generation ARBs before the 2006–2008 publications of clinical-trial results. During the period between the publication of the clinical-trial results and the scandals, first-generation ARBs were prescribed commonly: We estimate that between 2006 and 2013, more than six million people in Japan received those drugs (approximately 50 DDDs/1,000 persons). Contributing to that large number were, most likely, commercial promotional activities,^20^ the prestige of the journals in which the results were published,^1^^,^^10^^,^^11^ and the fact that first-generation ARBs appeared not only to lower blood pressure but also to protect against cardiovascular events.

Had the results of the clinical trials been true, a sustained preference for the drug (or at least a neutral view of it) might have been considered reasonable by healthcare professionals, patients and their families, and society at large. However, once the fabrication and falsification of data became widely known,^19^ and once the articles reporting the JIKEI-HEART, KYOTO-HEART, and KYOTO-HEART studies were retracted,^14^^–^^18^^,^^34^ there was a loss of social trust.^20^ Around the same time, another first-generation ARB-related scandal occurred—this one involving an inappropriate promotional activity for candesartan. The conflict of interest that was part of that inappropriate activity was also widely reported in the media.^8^ This series of scandals intensified the public demand for relationships between pharmaceutical companies and researchers to be made more transparent. It would certainly not be surprising for these events to result in negative views of first-generation ARBs among physicians and patients, which likely explains the accelerated decrease noted in this study.

Previous studies have assessed the effects of positive^3^ and negative news on drug use,^35^ and the present results are generally consistent with the results of those studies. To the best of our knowledge, this study is the first documentation to assess drug usage following both the publication of clinical-trial results and the associated scandals by a quasi-experimental design of ITSA, which is a robust approach to assess population-level interventions. After the scandals erupted, the 7-years-previous increase in the prescription of first-generation ARBs was overwhelmed and quickly reversed. One consequence of the scandals involving first-generation ARBs might have been a switch in prescribing, from first-generation to second-generation ARBs. In fact the use of second-generation ARBs gradually increased during the entire period under study, and not long after the scandals the use of second-generation ARBs exceeded that of first-generation ARBs (eFigure 1).

To understand why the effect of positive news was smaller than that of negative news, it is worth noting that the positive news was known almost exclusively within the medical community, whereas the negative news was also reported extensively via mass media and became widely known among the general public. Furthermore, given the fact that the costs of first-generation ARBs were covered under the national health insurance system of Japan, the scandals seen as a breach of public trust.

The main strength of this study is that it took advantage of a natural experiment. It was feasible because the costs of all antihypertensive drugs were covered under the universal healthcare-insurance system of Japan and the JPM database maintains information on the nationwide sales of first-generation ARBs. In fact, it is the only available source of this information to assess the impact of population-level interventions on nationwide drug use. In addition, rather than being phenomena that developed gradually, both the publication of the clinical trials’ results and the subsequent scandals were nearly-discrete events given the time scale under consideration, which made analyses of interrupted time series appropriate for these data. We also note that the interrupted time series design avoids problems caused by differences in individual-level patient characteristics, which is usually a major confounding factor in observational studies.^36^

Our study had several limitations. First, we only analyzed first-generation ARBs as a class, and thus used summary information on three agents, namely valsartan, candesartan, and losartan. Restrictions on the use of the JPM database prevented us from assessing the use of each drug agent individually. However, the population-level exposures did apply to the two main first-generation ARBs (valsartan and candesartan) at the same time. Thus, we could assess the effects of those exposures on the use of first-generation ARBs. Because losartan use in Japan was very low (less than half of that of comparable drugs), its inclusion in these data is unlikely to have compromised the study’s internal validity. Second, the interrupted time series analysis could have been affected by external events occurring during the time in question. One such external event might be publication of clinical trials in other countries. During the follow-up period (April 2005 through March 2017), the ONTARGET trial was published in the *New England Journal of Medicine* (in March 2008),^37^ which might have affected the use of first-generation ARBs in Japan. A meta-analysis was published in the *Lancet Oncology* in June 2010, reporting that the use of ARBs was associated with a slightly higher risk of cancer.^38^ Nonetheless, the results of clinical trials conducted in Japan may be more likely to be used for local drug-promotion activities. In the ITSA, another external event that might have affected the use of first-generation ARBs was the introduction of second-generation ARBs (olmesartan and telmesartan). Because that occurred before the start of the follow-up period, we were not able to assess its impact on the use of first-generation ARBs. Data published by Japan’s government show that utilization of first-generation ARBs increased before and after the introduction of second-generation ARBs.^39^ Third, we analyzed country-level aggregated drug data, which could not be linked to data from other sources. Therefore, we could not assess the effects of drug use on health outcomes. Fourth, it was not possible to tell whether the change in drug use was due to patients’ reluctance caused by media coverage or to physicians’ decisions caused by the retractions of key, high-profile papers. Media coverage may also have affected physicians’ decisions. Another limitation of the database is that drug-usage data were not directly measured, but were estimated from drug-sales data that were collected through wholesalers. Thus, our estimates were of county-level aggregated drug usage, based on the total sales in Japan and the DDD. To the extent that errors in those estimates occurred randomly, they would decrease the precision of our estimates of drug usage, but would not introduce bias. Considering those limitations, it should be noted that we assessed associations, not causation, even with the interrupted time series design, which is robust in the presence of confounding.^25^

This ITSA using nationwide drug-utilization data in Japan yielded two important findings: (1) the scandals were associated with decrease in the use of first-generation ARBs, and (2) that decrease was greater than the increase associated with the publication of “successful” clinical trials, making the net effect not zero but negative. The case of first-generation ARBs in Japan illustrates not only the absolute dependence of evidence-based medicine on honesty and scientific integrity but also the consequences of betraying the trust that is implicit in the social and healthcare-insurance context of Japan. Going a step further, to the extent that new drugs and other improvements in medical care are generally adopted slowly and cautiously, whereas reactions to unethical behavior and breaches of trust are quick and strong, these lessons from Japan could well be relevant worldwide.

## APPENDIX A. SUPPLEMENTARY DATA

eTable 1. Drug name, ATC, and DDD

eTable 2. Clinical trials of first-generation ARBs in Japan other than those referred to in the main text

eTable 3. The five models tested for the interrupted time series analysis

eTable 4. Methodological and reporting recommendations for interrupted time series studies

eMaterials 1. Description of the model used for interrupted time series analyses

eMaterials 2. Effects of the two exposures on the use of first-generation ARBs, with calcium-channel blockers rather than ACE inhibitors as the “control”

eFigure 1. Use of second-generation ARBs from April 2005 through March 2017
