# Supplementary material for: Changes in Drug Utilization After Publication of Clinical Trials and Drug-Related Scandals in Japan: An Interrupted Time Series Analysis, 2005–2017
Source: J Epidemiol. 2021 Jul 5;31(7):410–6. doi: 10.2188/jea.JE20200181 (PMC8187611; doi:10.2188/jea.JE20200181)
Supplement: Supplementary file 1 [file je-31-410-s001.pdf]

Supplementary material

eTable 1. Drug name, ATC, and DDD

eTable 2. Clinical trials of first-generation ARBs in Japan other than those referred to in the main text

eTable 3. The five models tested for the interrupted time series analysis

eTable 4. Methodological and reporting recommendations for interrupted time series studies

eMaterials 1. Description of the model used for interrupted time series analyses

eMaterials 2. Effects of the two exposures on the use of first-generation ARBs, with calcium-channel blockers rather than ACE inhibitors as the "control"

eFigure 1. Use of second-generation ARBs from April 2005 through March 2017

eTable 1. Drug name, ATC, and DDD

Drug name	ATC	DDD
Losartan	C09CA01	50 mg
Valsartan	C09CA03	80 mg
Irbesartan	C09CA04	0.15 g
Candesartan	C09CA06	8 mg
Telmisartan	C09CA07	40 mg

ATC, Anatomical Therapeutic Chemical Classification; DDD, defined daily dose.

eTable 2. Clinical trials of first-generation ARBs in Japan other than those referred to in the main text

Trial name	Trial's purpose and conclusion	Publications, promotional use, and scandals
**SMART**[a]	In this trial, the researchers aimed to examine the effect of ***valsartan*** on microalbuminuria in hypertensive patients with diabetes. The researchers wrote "We conclude that ARBs[a] can therefore be a first-line drug for the patients with type 2 diabetes and microalbuminuria."	**Publication:** The results were presented at ISH2006,[d] and they were published in *Diabetes Care* in June 2007.<br>**Promotion:** After ISH2006, the results were used for commercial promotional activities.<br>**Scandal:** Because of fabricated data, the published report was retracted in March 2014.
**VART**[b]	In this trial, the researchers aimed to examine the effect of ***valsartan*** on cardiovascular disease in hypertensive patients. The researchers wrote "Therefore, although BP levels were well controlled and remained equal in the two groups, valsartan had more protective effects on the heart and kidney than amlodipine in Japanese hypertensive patients."	**Publication:** The results were published in *Hypertension Research* in October 2010.<br>**Promotion:** After they were published, the results were used for commercial promotional activities.<br>**Scandal:** Because of fabricated results, the published report was retracted in November 2016.
**Nagoya Heart**[c]	In this trial, the researchers aimed to examine the effect of ***valsartan*** on cardiovascular disease in hypertensive patients with glucose intolerance. The researchers wrote "Composite cardiovascular outcomes were comparable between the valsartan- and amlodipine-based treatments in Japanese hypertensive patients with glucose intolerance."	**Publication:** The results were published in *Hypertension* in January 2012.<br>**Promotion:** After they were published, the results were used for commercial promotional activities.<br>**Scandal:** Because of fabricated results, the published report was retracted in August 2018.

[a]SMART: The Shiga Microalbuminuria Reduction Trial
[b]VART: The Valsartan Amlodipine Randomized Trial
[c]Nagoya Heart: Comparison Between Valsartan and Amlodipine Regarding Cardiovascular Morbidity and Mortality in Hypertensive Patients With Glucose Intolerance

[d]ISH2006: The 21st Scientific Meeting of the International Society of Hypertension, which was held in October of 2006, in Fukuoka, Japan.

eTable 3. The five models tested for the interrupted time series analysis

Model	Number of parameters	QIC
**1) Changes in level and changes in slope, with seasonality adjustment via calendar-month indicators**<br>**Correlation structure: independent**<br>**This model is considered to be the best, because it had the lowest QIC.**	**22**	**3231074.34**
2) Changes in level and changes in slope, with seasonality adjustment via calendar-month indicators<br>Correlation structure: exchangeable	Did not converge	
3) Changes in level and changes in slope, with seasonality adjustment via calendar-month indicators<br>Correlation structure: autoregressive	22	3231098.84
4) Changes in level, with seasonality adjustment via calendar-month indicators<br>Correlation structure: independent	18	4166671.60
5) Changes in level and changes in slope, with seasonality adjustment via Fourier transformation<br>Correlation structure: independent	15	7663481.71

eTable 4. Methodological and reporting recommendations for interrupted time series studies

Item	Item no	Recommendation	Where to look in this report, if applicable
Title and abstract	1	Indicate the study design (interrupted time series) in the title or abstract.	ABSTRACT
Introduction			
Background	2	Provide background regarding the intervention and setting under investigation to support the study.	The first, second, and third paragraphs of the Introduction section.
Objectives	3	(a) State specific objectives and any pre-specified hypotheses.<br>(b) Distinguish between primary and secondary objectives.	(a) The last paragraph of the Introduction section.<br>(b) The last paragraph of the Introduction section. We had a primary objective only (no secondary objective): to compare the change in the use of first-generation ARBs after the trials' results were published to the change after the scandals occurred.
Methods			
Intervention	4	Define the intervention time point(s) used in the analysis.	The section called "*Period of exposures to clinical trial publications and subsequent scandals*".
Participants	5	(a) List eligibility criteria and methods of selection.<br>(b) Define subgroups.<br>(c) Consider including a comparison group not exposed to the intervention as a secondary group of participants.	(a) The section called "*Use of first-generation ARBs*".<br>(b) Not applicable. (We could not conduct subgroup analyses of data on individual drugs due to restrictions on the use of the database.)<br>(c) The first paragraph of the section called "*Statistical analysis*" (We used ACE inhibitors as a comparison group in the main analysis.)
Data sources and	6	(a) List data source(s).	(a) The section called "*Design, setting, and data source*".

measurement		(b) Comment on data completeness, validity, and changes in data coverage over time.	(b) The section called "*Design, setting, and data source*".
Variables	7	(a) Define all variables.<br>・Outcome variable(s).<br>・Descriptive and stratifying variable(s).<br>(b) Comment on change in variable coding over time.<br>(c) Consider including details of variable coding in Supplementary material, for example, appendix or research Web site.	・Outcome variable(s): The section called "*Use of first-generation ARBs*".<br>・Descriptive and stratifying variable(s): Not applicable<br>(b) The section called "*Use of first-generation ARBs*" and Supplementary Table 1.<br>(c) Supplementary Table 1.
Statistical methods	8	(a) Report all statistical methods.<br>・Study time intervals, for example, monthly, quarterly.<br>・Regression model, for example, ARIMA, linear, segmented.<br>For ARIMA models, indicate the intervention function, for example, point, ramp, or step.<br>Indicate the appropriateness of linear model(s) when applied.<br>・Number of preintervention, postintervention, and between intervention data points.<br>(b) Define the study period and number of preintervention data points used in forecasting.<br>(c) Indicate how autocorrelation, nonstationarity, and	(a) The section called "*Statistical analysis*".<br>・Study time intervals, for example, monthly, quarterly: The first sentence in the section called "*Statistical analysis*".<br>・Regression model, for example, ARIMA, linear, segmented: The first paragraph in the section called "*Statistical analysis*"; Supplementary Text 1; Supplementary Table 3.<br>・Number of preintervention, postintervention, and between intervention data points: Table 2.<br>(b) The section called "*Design, setting, and data source*"; Table 2.<br>(c) The first paragraph in the section called "*Statistical analysis*".<br>(d) The section called "*Period of exposures to clinical trial publications and to subsequent scandals*".<br>(e) The second and third paragraph of the section called

		seasonality were tested and handled.<br>(d) Consider a lag period if intervention effects are gradual or delayed.<br>(e) Define and distinguish between primary and secondary or sensitivity analyses.<br>(f) Consider use of comparison outcome(s) and/or population(s) not exposed to the intervention(s) as secondary analyses.<br>(g) Report statistical software used for analysis.	"*Statistical analysis*".<br>(f) We used ACE inhibitors and calcium-channel blockers as the comparison groups.<br>(g) The last paragraph in the section called "*Statistical analysis*".
Results			
Participants	9	(a) Report the number of individuals and/or observations in each group analyzed.<br>(b) Consider use of a flow diagram.<br>(c) Describe characteristics and indicate missing data.	(a) Not applicable to aggregated drug data.<br>(b) Not applicable to aggregated drug data.<br>(c) Not applicable to aggregated drug data.
Outcome data	10	(a) Report the number of outcomes examined over the study period.<br>(b) Report the average, minimum, and maximum number of outcomes across time intervals.<br>(c) Report on data variability.<br>(d) Comment on outliers and ceiling or floor effects where relevant.	(a) Figure 1.<br>(b) Figure 1.<br>(c) Figure 1.<br>(d) Not applicable.
Main results	11	(a) Present results using a graphical display with intervention time point(s) clearly defined.	(a) Figure 1.<br>(b) Figure 1.

		(b) Consider including forecasted results graphically.<br>(c) Report absolute and/or relative change(s) and their significance, for example, clinical or policy and statistical.	(c) The first paragraph of the Results section.
Other analyses	12	Report additional results (secondary and sensitivity analyses) in the article, appendix, or research Web site.	Supplementary Tables 3-4.
Discussion			
Key results	13	Summarize key results with reference to study objectives.	The first paragraph of the Discussion and Conclusions section.
Context	14	(a) Provide context related to possible confounding.<br>・Discuss relevant cointerventions that occurred during the study period.<br>・Comment on the stability of participant characteristics over time.<br>・Comment on the stability of outcome coding over time.<br>(b) Discuss results of comparison analyses or provide a rationale if no comparison group was considered.	(a) The sixth and seventh paragraph of the Discussion and Conclusions section.<br><br>(b) The last paragraph of the Results section.
Limitations	15	(a) Discuss limitations of the study.<br>(b) Comment on data variability and appropriateness of the number of data points.<br>(c) Comment on ceiling or floor effects and outliers	(a) The seventh paragraph of the Discussion and Conclusions section.<br>(b) The second paragraph of the Results section.<br>(c) Not applicable.

		where relevant.<br>(d) Discuss direction and magnitude of any potential bias.	(d) The seventh paragraph of the Discussion and Conclusions section.
Interpretation	16	Provide overall interpretation of results considering objectives, limitations, results from similar studies, and other relevant evidence.	The last paragraph of the Discussion and Conclusions section.
Other information			
Funding	17	List funding source(s) and role of funders.	The last paragraph of the Methods section.
References	18	Reference methodological articles that support statistical methods used.	References 25, 26, 30, 31, and 32.

ARIMA, autoregressive integrated moving average; GEE, generalized estimating equation.

Items adapted from the Strengthening the Reporting of Observational Studies in Epidemiology statement.

eMaterials 1. Description of the model used for interrupted time series analyses

$$\ln(N_t) = \ln(W_t) + \beta_0 + \beta_1 Time_{0t} + \beta_2 Publication_t + \beta_3 Publication_t \times Time_{1t}$$
$$+\beta_4 ARB + \beta_5 ARB \times Time_{0t} + \beta_6 ARB \times Publication_t$$
$$+\beta_7 ARB \times Publication_t \times Time_{1t} + \beta_8 Scandal_t$$
$$+\beta_9 Scandal_t \times Time_{2t} + \beta_{10} ARB \times Scandal_t + \beta_{11} ARB \times Scandal_t \times Time_{2t}$$
$$+\beta_{12} Month2_t + \beta_{13} Month3_t + \cdots + \beta_{22} Month12_t + e_t$$

The model included two exposure variables ($Publication_t$ and $Scandal_t$); three time variables (the time elapsed since the start of the study [$Time_{0t}$], the time elapsed since the publications [$Time_{1t}$], and the time elapsed since the scandals [$Time_{2t}$]); an indicator variable of first-generation ARBs compared with the comparison group (*ARB*); variables representing interactions between the exposure variables, the ARB indicator variable, and the time variables; and dummy variables for the calendar months.

The exposure variable $Publication_t$ was 0 when *t* was October 2006 or earlier and 1 when *t* was April 2007 or later. The exposure variable $Scandal_t$ was 0 when *t* was February 2013 or earlier and 1 when *t* was August 2013 or later. The variable N_t indicates the number of patients receiving first-generation ARBs at time *t*. The variable W_t indicates the total number of residents in Japan at time *t*. The term e_t indicates the residual at time *t*.

eMaterials 2. Effects of the two exposures on the use of first-generation ARBs, with calcium-channel blockers rather than ACE inhibitors as the "control"

With calcium-channel blockers rather than ACE inhibitors used as the "control", the results were almost exactly the same as those reported in the main text: Publication of the clinical-trial results was associated with an increase in the use of first-generation ARBs (before-to-after ratio of DDDs/1,000 persons, 1.11; 95% confidence interval [CI], 1.11-1.12). In contrast, the scandals were associated with a decrease (before-to-after ratio of DDDs/1,000 persons, 0.81; 95% CI, 0.81-0.82). Before the results of the clinical trials were published, the use of first-generation ARBs had been increasing (annual change in DDDs/1,000 persons/year, 1.10; 95% CI, 1.09-1.12). There was little change between the time of the trials' publication and the scandals (annual change in DDDs/1,000 persons/year, 0.99; 95% CI, 0.99-0.99). Once the scandals erupted, the use of first-generation ARBs decreased (annual change in DDDs/1,000 persons/year, 0.93;95% CI, 0.93-0.93). The net effect of the two exposures was a 10% decrease in the use of first-generation ARBs (DDDs/1,000 persons, 0.90; 95% CI, 0.83-0.85).

eFigure 1. Use of second-generation ARBs from April 2005 through March 2017

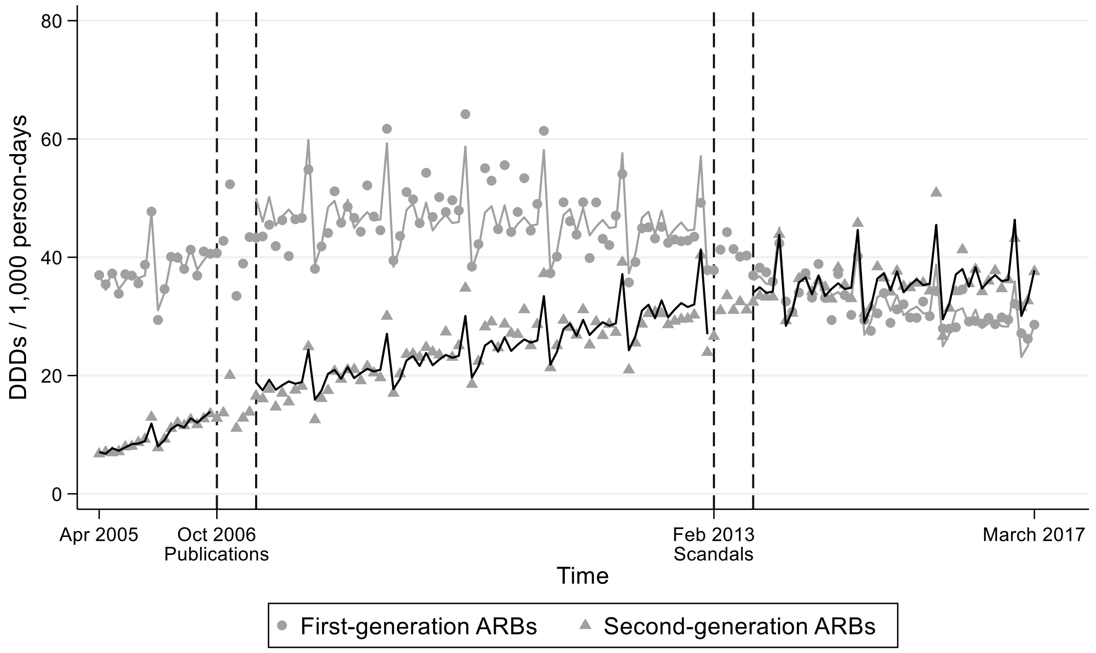

The use of second-generation ARBs increased gradually, and after the scandals it exceeded that of first-generation ARBs
